# Iranian Smell Diagnostic Test in Covid-19 Disease; Report of Covid-19 Center of North of Iran

**DOI:** 10.22088/cjim.13.0.204

**Published:** 2022

**Authors:** Shayan Alijanpour, Payam Saadat, Mehran Shokri, Sepanta Saadat, Azam Khodami

**Affiliations:** 1 Student Scientific Research Center, School of Nursing and Midwifery, Tehran University of Medical Science, Tehran, Iran; 2 Education, Research and Planning Unit, Pre-hospital Emergency Organization and Emergency Medical Service Center, Babol University of Medical Science, Babol, Iran; 3 Mobility Impairment Research Center, Health Research Institute, Babol University of Medical Sciences, Babol, Iran; 4 Infectious Diseases and Tropical Medicine Research Center, Health Research Institute, Babol University of Medical Sciences, Babol, Iran; 5 Student Research Committee, Zahedan University of Medical Science, Zahedan, Iran; 6 Ayatollah Rouhani Hospital, Babol University of Medical Sciences, Babol, Iran

**Keywords:** COVID-19, Olfaction Disorders, Coronavirus, Coronavirus Infections.

## Abstract

**Background:**

SARS-CoV-2 is a pandemic coronavirus that causes the COVID-19 syndrome. In the pandemic of COVID-19 many patients were affected to new onset olfactory dysfunction. Since there is a dearth of research studies regarding the standard smell test, the present study was conducted to fill this gap.

**Methods:**

The present retrospective cohort study was conducted on 250 clients with or without diagnosis of Covid-19 disease who referred to Covid-19 centers of North of Iran. Two groups were matched for age and sex. Data were collected by examination, demographic and clinical information questionnaire and Iranian smell diagnostic test. The binary logistic regression to estimate the odds ratio value in SPSS version 23.0 was used.

**Results:**

One-hundred cases (42.2%) had hyposmia and 20 cases (8.4%) were found to have anosmia. Type of covid-19 sign and symptom were statistically significant with olfactory dysfunction (41 cases, 31.8%), fever (28 cases, 21.7%), weakness and dyspnea (15 cases, 11.6%), (p=0.0001). The urban residency equal OR=6.42 (3.04-13.53) to rural residency for olfactory dysfunction (p=0.0001). Covid-19 patients’ OR=61.25 (27.36-137.11) chance to be affected by the olfactory dysfunction in compare to control group (p=0.0001). Also, with increasing age, chance of olfactory dysfunction changed from OR=0.61(1.16-0.13) to OR=1.89 (0.82-4.33). Furthermore, female chance OR=1.21 (0.72-2.03) and employee patients was OR=2.29 (1.30-4.04) to olfactory dysfunction.

**Conclusion:**

Alf of the patients were affected by olfactory dysfunction. Furthermore, Covid-19 patients, urban residency, lower age, female and employee were the prognostic factors for olfactory dysfunction. The standard olfactory tests such as IR-SIT is suggested for screening and detecting the clients probably affected by covid-19 especially in younger ages.

SARS-CoV-2 (CoV-2) creates the COVID-19 syndrome as a pandemic disease(1).This challenge has been observed in more than 1.5 million patients globally as of 29 June 2021(2). One of the countries which is involved in pandemic (after China) is the Islamic Republic of Iran with 3192809 laboratory-confirmed test and 84127 total deaths up to June 30, 2021(3). The study on these patients showed that 36.4% of them had a variety of neurological symptoms and damages (4). The movement into the brain through the cribriform plate near the olfactory bulb could be a route that would enable the virus to enter and influence the brain. Thus, the findings such as olfactory dysfunction in an early stage of clients should consider an assessment for central nervous system involvement(2, 5). Also, in study on 2,428 patients with new-onset anosmia during the COVID-19 pandemic it was revealed that 16% of them had anosmia as an isolated symptom(6). Coronavirus can disrupt ciliary nasal epithelium, which plays a significant role in the mechanism of olfactory dysfunction (OD).

Also, olfactory epithelial cells express the CoV-2 receptor, angiotensin-converting enzyme 2 (ACE2); yet, the precise cellular subtype that may mediate anosmia in COVID-19 remains unclear (7). In the medical literature, COVID-19-related anosmia is a new terminology. Half of the clients with COVID-19 were affected by anosmia. In more than 80% of cases, it is associated with dysgeusia (8). These clients are exposed to injury because of dysfunction due to smelling dangerous odors such as gas or spoiled food. Furthermore, nutritional and psychiatric status can be altered(9). Academy of Otolaryngology of America suggests that olfactory dysfunction should be used in screening of COVID-19 clients(10). 

For this work, standard criteria seem to be useful. Due to limited studies which used standard criteria vs. many studies which use questionnaire and self-report for screening the olfactory dysfunction, the present study was conducted. The purpose of this study is to assess the olfactory dysfunction status in COVID-19 clients with standard Iranian Smell Identification Test (IR-SIT). To the best of our knowledge, this study is the first survey with standard test for screening the OD in the north of Iran.

## Methods

**Study Population: **This retrospective cohort study was conducted on 250 clients with covid-19 who referred to COVID-19 center (Ayatollah Rouhani Hospital) of Babol, Mazandaran, North of Iran, during the years 2020-2021 after approval by Ethics committee of Babol University of Medical Sciences (IR.MUBABOL.HRI.REC.1399.095). The informed written consent was obtained from patients to participate in the study. 

The control group included individuals without covid-19 disease in screening test who referred to other departments of Ayatollah Rouhani Hospital of Babol. Two groups matched based on the age and sex. Inclusion criteria included patients aged 15 or upper that consented to participate to current study. After specialist examination, "COVID-19 detection protocol" of National Ministry of Health was used which includes PCR test or CT imaging with positive ﬁndings to enroll patients in the present study. The risk factor for OD such as head trauma, nasal or sinus diseases (polyps, rhinitis, rhinosinusitis, rhinoplasty), neoplasm of nasal or brain, smoking, neurodegenerative diseases (Alzheimer, Multiple sclerosis, Epilepsy, etc.), mental disorders (Schizphrernia, Depression, etc.), recent adult cold, migraine, pregnancy and exposure to chemical substances were excluded. Demographic information with clinical parameters such as past surgery history, family history of olfactory dysfunction, time of admission, clinical manifestation, screening place of covid-19 were also recorded. Age and sex were matched in two groups. 

**Assessment of olfactory function: **The case and control groups were assessed by IR-SIT that was adopted by the efforts of Taherkhani et al (15). This well-validated quick IR-SIT determines normosmia, mild hyposmia, severe hyposmia and anosmia (11) by 6 odors (banana, rosewater, cinnamon, garlic, mint, and melon) as sticker with high accuracy in differentiating anosmia, hyposmia and normosmia. The researcher had the clients smell the scratched sticker form 2-cm distance and select from the 4-alternative test sheet. Clients with 5 or 6 correct answers were considered normosmia, 1 to 4 hyposmia, and zero anosmia(12).

**Statistical Analysis: **We used Pearson correlation coefficient, Chi-square, t-test and Binary logistic regression to estimate the odds ratio (OR) value in SPSS version 23 and p ***< ***0.05 was set as the significant level.

## Results

After inclusion and exclusion criteria, 240 patients were enrolled in the current study. The age and sex were matched in two groups. The mean age in control and COVID-19 groups was 51.13±12.81, 51.95±16.66, respectively. Distribution of gender was 104 (43%) males vs. 136 (56.7%) females. Fifty-two males (50%) were both in control and COVID-19 groups vs. 68 females (50%) were in two groups. Table 1 shows the demographic characteristics of COVID-19 and control groups. One- hundred thirty-six cases (56.7%) were female and the mean age was between 51.54±14.84 years. 205 patients (86.5%) were residents of Babol and 32 cases (13.5%) lived in other cities(p=0.17). 

Regarding the admission time 64 cases (58.2%) referred to the hospital in the evening, 24 cases (21.8%) in the morning and finally 22 cases (20%) were admitted at night(p=0.09). The chief symptoms reported (41 cases, 37.3%) were fever and weakness (33 cases, 30%). The mean day period of fever, as the main symptom of the disease, was 10±16.66, weakness, as the second main symptom, was 7.5±5.78 and myalgia was 3.5±2.12.

After OD test, 117 patients (49.4%) were diagnosed as normal, 100 cases (42.2%) had hyposmia and 20 cases (8.4%) suffered from anosmia. The relationship between the type of covid-19 symptoms and the OD was statistically significant with 41 cases (31.8%) having fever, 28 cases (21.7%) having weakness and 15 cases (11.6%) having dyspnea (p=0.0001). As shown in Table 2, the difference in age, marital status, education, occupation and residential status was statistically significant between the two groups (p<0.05).

As shown in Table 3, after olfactory test, patients were divided into normal and OD (hyposmia and anosmia). In binary regression, logistic results showed that urban residency (OR=6.42, 3.04-13.53) equal to rural residency for olfactory dysfunction (p=0.0001). Covid-19 patients with OR=61.25(27.36-137.11) had a high of chance of being affected by the OD in comparison with the control group (p=0.0001). Also, with increasing age from middle age to elderly the chance of OD changed from OR=0.61(1.16-0.13) to OR=1.89 (0.82-4.33). Furthermore, female chance to get olfactory dysfunction was OR=1.21 (0.72-2.03). Also, difference in job showed that the chance of employee patients was OR=2.29 (1.30-4.04) (p=0.004) to non-salary patients while the chance of freelance work was OR=0.64 (0.28-1.47) in comparison with the non-salary patients (p=0.3).

**Table 1:** Frequency distribution of demographic characteristics in covid-19 and control groups

**Variable**	**category**	**Olfactory function**			**Total**	**P value**
		**Normal (%)**	**Hyposmia (%)**	**Anosmia (%)**		
**Gender**	male	48(46.6)	44(42.7)	11(10.7)	103(43)	0.50
	female	69(51.5)	56(41.8)	9(6.7)	134(57)	
**Age (year)**	14-40	24(45.3)	79(57.2)	14(30.4)	53(22.3)	0.004
	40-64	28(52.8)	46(33.3)	26(56.5)	138(58.2)	
	65 to upper	1(1.9)	13(9.4)	6(13)	46(19.5)	
**Marital status**	Married	101(47)	94(43.7)	20(9.3)	215(90.7)	0.050
	Single	9(60)	6(40)	0(0)	15(6.3)	
	Divorced	7(100)	0(0)	0(0)	7(3)	
**Education**	Under diploma	40(37.7)	54(50.5)	13(12.1)	107(45)	0.007
	Diploma	29(51.8)	23(41.1)	4(7.1)	56(24)	
	Upper diploma	48(69.4)	23(31.1)	3(4.1)	74(31)	
**Occupation**	Without salary	40(34.2)	55(55.6)	13(65)	108(46)	0.004
	employed	23(19.7)	11(11.1)	0(0)	34(14)	
	freelance	54(46.12)	33(33.3)	7(35)	94(40)	
**Residential status**	Rural	10(18.2)	36(65.5)	9(16.4)	55(23)	0.0001
	Urban	107(58.8)	64(35.2)	11(6)	182(77)	
**Cardiovascular disease**	no	3(4.3)	49(70)	18(25.7)	70(90)	0.19
	yes	0(0)	8(100)	0(0)	8(10)	
**Diabetes mellitus**	no	2(3)	49(73.1)	16(23.9)	67(86)	0.59
	yes	1(9.1)	8(72.7)	2(18.2)	11(14)	
**Diagnose of Covid-19**	clinic	1(4.3)	16(69.6)	6(26.1)	23(20)	0.36
	hospital	10(11.1)	66(73.3)	14(15.6)	90(80)	
**Group**	control	105(87.5)	15(12.5)	0(0)	120(50.6)	
	Test	12(10.3)	85(72.6)	20(17.1)	117(49.4)	0.0001

**Table 2:** Evaluation of rapid Iranian olfactory Test in covid-19 disease and control group

**Variable**	**Category**	Sense of Smell	**Covid-19 (%)**	**Control(%)**	**Total(%)**	**p-value**
**Age (year)**	14-40	Olfactory dysfunction	27(93.1)	2(6.9)	29(54.7)	0.0001
		Normosmia	6(25)	18(75)	24(45.3)	
	40-65	Olfactory dysfunction	5(6.3)	8(13.6)	79(57.2)	
		Normosmia	5(6.3)	74(93.7)	79(57.2)	
	65 to upper	Olfactory dysfunction	27(84.4)	5(15.6)	32(69.6)	
		Normosmia	1(7.1)	13(92.9)	14(30.4)	
**Marital status**	Married	Olfactory dysfunction	100(87.7)	14(12.3)	114(53)	0.0001
		Normosmia	12(11.9)	89(88.1)	101(47)	
	Bachelor	Olfactory dysfunction	5(83.3)	1(16.7)	6(40)	
		Normosmia	0(0)	9(100)	9(60)	
	Divorced	Olfactory dysfunction	0(0)	0(0)	0(0)	
		Normosmia	7(100)	0(0)	7(100)	
**Education**	Non-diploma	Olfactory dysfunction	57(85.1)	10(14.9)	67(62.6)	0.0001
		Normosmia	6(15)	34(85)	40(37.4)	
	Diploma	Olfactory dysfunction	27(100)	0(0)	27(100)	
		Normosmia	3(10.3)	26(89.7)	29(51.8)	
	Upper diploma	Olfactory dysfunction	21(80.8)	5(19.2)	26(35.1)	
		Normosmia	3(6.3)	45(93.8)	48(64.9)	
**Occupation**	Without salary	Olfactory dysfunction	63(92.6)	5(7.4)	68(63)	0.0001
		Normosmia	6(15)	34(85)	40(37)	
	Employed	Olfactory dysfunction	8(72.7)	3(27.3)	11(32.4)	
		Normosmia	1(4.3)	22(95.7)	23(67.6)	
	Freelance	Olfactory dysfunction	33(82.5)	7(17.5)	40(42.6)	
		Normosmia	5(9.3)	49(90.7)	54(57.4)	
**Residential status**	urban	Olfactory dysfunction	61(81.3)	14(18.7)	75(41.2)	0.003
		Normosmia	6(5.6)	101(94.4)	107(58.8)	
	rural	Olfactory dysfunction	44(97.8)	1(2.2)	45(81.8)	
		Normosmia	6(60)	4(40)	10(18.2)	

**Table 3:** Binary Regression Logistic In Covid-19 and Control Groups

**Variable**	**Crude**			**adj**		
	**OR**	** 95 CI for OR**	**P**	**OR**	**95 CI for OR**	**P**
Test group	61.25	27.36-137.11	0.0001	71.26	24.99-203.14	0.0001
Residential status	6.42	3.04-13.53	0.0001	1.33	0.36-3.52	0.83
Gender	1.21	0.72-2.03	0.45	1.77	0.63-4.99	0.27
non-salary	-	-	0.001	-	-	0.68
employee	2.29	1.30-4.04	0.004	1.61	0.54-4.76	0.38
freelance	0.64	0.28-1.47	0.3	1.23	0.34-4.43	0.74
adult	-	-	0.007	-	-	0.07
Middle age	0.61	0.32-1.16	0.13	1.42	0.48-4.16	0.51
old	1.89	0.82-4.33	0.13	4.44	1.12-17.55	0.03

## Discussion

Covid-19 as a pandemic disease has rapidly spread all over the world. OD is one of the complications of covid-19 that has been considered by researchers, recently. The result of the present study revealed that 50.6% of patients were affected by olfactory dysfunction, 100 cases (42.2%) had hyposmia, and 20 cases (8.4%) suffered from anosmia. In Saniasiaya et al.’s meta-analysis, OD was observed in 54.40% of European and 31.39% of Asian COVID-19 patients(13). In Klopfenstein et al.’s study, 54 COVID-19 patients (47%) had anosmia. Also, 67% of the hospitalized cases were female and 37% were male(8). Kaye et al. found anosmia in 73% of the subjects prior to COVID-19 diagnosis and it was the initial symptom in 26.6%(14) of the cases. In Lechien et al.’s study, 86% of mild-to-moderate COVID-19 disease patients had anosmia and in 11.8% cases it started before other symptoms (6). Bayesian et al., reported that in 70.2% of clients, anosmia was a key symptom in COVID-19 (6). 

In Tabari et al.’s study where IR-SIT was used to detect the OD in Iran, it was found that 60% of the patients with COVID-19 had hyposmia(12). On the other hand, Mao et al.’s study reported that 5% of the patients had hyposmia (15). The results of various studies are different and the gap between them can be attributed to some factors. First, some clients cannot diagnose hyposmia, especially older individuals. so, self-reported study of anosmia or hyposmia may be unreliable in detection(16). Second, studies showed that the methodology used to investigate the olfactory function had a deep impact on smell performance prevalence rate identification: the pooled prevalence estimate of smell loss was 77% when assessed through objective measurements and 45% with subjective measurements, suggesting that subjective measures may underestimate the true prevalence of smell loss(17). Third, anosmia is more detectable compared to hyposmia (12). Therefore, many conﬁrmed clients may not report hyposmia. With this regards, performing a standard OD test vs. self-report for covid-19 is desirable.

The relationship between type of covid-19 symptoms and OD was statistically significant with41 cases (31.8%) having fever, 28 cases (21.7%) having having weakness and 15 cases (11.6%) dyspnea. In Borah et al.’s study, the most common symptoms were fever (93%) and cough (85%). The malaise, generalized body ache and abdominal symptoms like diarrhea were the least common symptoms. The most common ear, nose, and throat (ENT) manifestations were sore throat (80%) and headache (76%). Also, hyposmia was seen in 44% of patients (18). In Ermis et al.’s study, hyposmia (26 %), pain and general muscle weakness (32%), and headache 21% were observed(19). Also, in Ermis et al.’s study, they were only able to neurologically characterize 38.4% of the patients. In Tabari et al.’s study, 28 cases (41%) had a normal sense of smell and 40 cases (59%) were found to have hyposmia by IR-SIT. The most common symptoms were cough 45(66%), dyspnea 40(58%), fever 37 (54%), myalgia 33(48%) and sore throat 17(25%). Fifty-eight percent of inpatients with COVID-19 were measured for OD and had hyposmia according IR-SIT test; 10 cases (25.0%) had severe smell dysfunction and 30 patients (75.0%) had mild to moderate OD, while there were no pure anosmic subjects. Generally, 60.7% of the patients who did not report hyposmia, by IR-SIT test were detected as having some degrees of OD. Inpatients with hyposmia under-reported their loss of sense of smell by 70% (12/68 self-report vs. 40/68 with objective olfactory testing) (12). 

In binary regression logistic result showed that urban residency equal OR=6.42 to rural residency for olfactory dysfunction. Covid-19 cases with an OR=61.25 had a higher chance to be affected by the OD compared to the control group. In D’Ascanio et al.’s study, COVID-19 patients showed an increased risk of olfactory dysfunction in comparison with the control groups ( OR, 77.2; P =0.003)(20). Also, with increasing age from middle age to elderly, the chance of OD changed from OR=0.61to OR=1.89. In Talavera et al.’s study, age is another significant confounder, given the fact that older age is associated with lower OD rates and poor outcomes(21). 

Siso-Almirall et al. concluded that multivariate analyses were conducted after adjusting for age and it was found that OD was a predictor of better outcomes (22). Tabari et al. reported that the mean age in normal sense patients was 43.32±14.74 vs. 44.43±17.20 in hyposmic patients which was not significant(12). In Carignan et al.’s study, the OR for the association of anosmia with SARS-CoV-2 positivity was 20.0(23). It seems that OD could be attributed to aging or covid-19, physiologic changes and the probability of worsening condition. On other hand, detection of OD in these patients is difficult. More surveys are recommended to elucidate whether the association between COVID-19 and OD outcomes is age dependent. 

Furthermore, female chance to get olfactory dysfunction was OR=1.21. In Andrews et al.’s study, the prognosis of OD among patients was negatively influenced by female sex (P = 0.02)(24). On the other hand, Carignan et al.’s study, showed that the difference between male and female was not significant(23). In D’Ascanio et al.’s study, women had no significant risk of smell loss compared to men (OR, 1.22; P = 0.8) (20). However, in Andrews et al.’s study, there was a male to female ratio of 1:3 which can affect this relation. On other hand, the total sample volum in D’Ascanio et al.’s study was 26 patients which is very low compared to other studies. In the present study, 134 subjects (57%) were female and the gender variable was matched between two groups. Also, difference in job showed that the chance of employee patients was OR=2.29 to non-salary patients and freelance work was OR=0.64 compared to non-salary patients. Andrews et al. study indicated that occupational role such as nurse or health care agent negatively influenced OD (P = 0.002)(24). It can be related to services provided by government staffs in covid-19 pandemic. Furthermore, some jobs were not closed in this period.

The main limitation of the present study was the small number of patients who cannot follow-up for during the current COVID 19 pandemic situation. On other hand, to our knowledge, the present study is the first standard smell test for COVID-19 patients in the north of Iran which is more sensitive instead of relying on self-reports or subjective questionnaires.

## Conclusion:

The result of the study revealed that half of the patients were affected by olfactory dysfunction. Furthermore, Covid-19 patients, urban residency, lower age, being female and an employee were the prognostic factors for olfactory dysfunction. The standard olfactory test such as IR-SIT is suggested especially in younger age to screen and detect the clients probably affected by covid-19.
